# The “UFO Taboo” Is What IR Theorists Make of It: “Sovereignty and the UFO” in Citational Perspective

**DOI:** 10.1177/03043754231219831

**Published:** 2023-12-02

**Authors:** Michael P. A. Murphy

**Affiliations:** 1 4257 Queen’s University, Kingston, ON, Canada

**Keywords:** international relations, sovereignty, scientometrics, actor-network theory, interpretive methods, academic publishing

## Abstract

In 2008, Alexander Wendt and Raymond Duvall published an article titled “Sovereignty and the UFO,” which demonstrated how a UFO taboo in international relations theory upheld an anthropocentric model of sovereignty. At a distance of a decade and a half, this review evaluates the validity of the claim that a UFO taboo exists in international relations, and explores the citational practices that influence the prestige economy of the field. The article employs a methodology of interpretive scientometrics informed by methodological debates in political science and international, as well as theoretical debates in actor-network theory. After testing the claim of the UFO taboo in a comparative perspective, the article investigates the strategies of association (weak and strong) present in the citations of “Sovereignty and the UFO.” In addition to a revaluation of core claims in an often-read but less-often-cited article in international relations theory, this article provides important insights into how citation works in the discipline of international relations.

## Introduction

UFO ignorance is not simply a gap in our knowledge, like the cure for cancer, but something actively reproduced by taboo…Thus, our puzzle is not the familiar question of ufology, “What are UFOs?” but, “Why are they dismissed by the authorities?” Why is human ignorance not only unacknowledged, but so emphatically denied? In short, why a **taboo**? These are questions of social rather than physical science, and do not presuppose that any UFOs are ETs. Only that they might be. (Wendt & Duvall, 2008, p. 611).

Sovereignty has long been one of the core concepts of international relations theory (Angell, 1914; Morgenthau, 1948; 1954; Waltz, 1979; Strange, 1996; Krasner, 1999), describing a key principle of states in world politics. Often drawing specifically from a European tradition of philosophy steeped in Christian political theology (Schmitt, 2005; Kantorowicz, 1957), sovereignty is taken as the crown of authority in polities. While the debate has highlighted different aspects of sovereignty, argued for its change or continuity, and suggested its waxing or waning importance, sovereignty has a conceptual staying power in the popular imaginary of theorists of international relations. Even among critical accounts that draw attention to the failures and harms of international relations theory, sovereignty often looms large (Georgis & Lugosi-Schimpf, 2021; Mack & Na’puti, 2019; Moreton-Robinson, 2015; Pourmokhtari, 2013; Shilliam, 2006; Weber, 2016). Whether supportive or critical, the discussion of sovereignty largely focused on human societies in the world, in line with longstanding anthropocentric tendencies in political theory and international relations.

Standing in stark contrast to the anthropocentric tendency of sovereignty theorizing is a 2008 article by Alexander Wendt and Raymond Duvall entitled “Sovereignty and the UFO,” which appeared in *Political Theory*.^1^ Wendt and Duvall identify an anthropocentric consensus despite bitter debates on the nature of sovereignty, and suggest that “anything that challenged anthropocentric sovereignty, it seems, would challenge the foundations of modern rule” (2008, 609). While hypothetical scenarios of the “Second Coming” and ecological considerations of animal rights are mentioned as potential thought experiments, they turn instead to the radical thesis of taking seriously the potential that unidentified flying objects (UFOs) might be extraterrestrial in origin. To do so, they challenge what they call the “UFO taboo” to understand how “the authoritative disregard for [UFOs] brings clearly into view the limits of anthropocentric metaphysics” (Wendt & Duvall, 2008, 609). While Wendt has from time to time invoked UFOs and alien life to illuminate different arguments about international relations theories and human life (e.g., Wendt, 1992, p. 405; 1999, pp. 73, 108; 2015, p. 269), the outer-spatial is not (yet?) a familiar level of analysis.^2^

What specifically do the authors mean when they—as we saw in the epigraph—call the UFO silence in international relations a *taboo*? They suggest that instead of merely not appearing in research, there is instead an “authoritative disregard of UFOs” that includes “active denial of their object status” through decrying UFOlogy as pseudoscience, active dismissal of public UFO claims, and maintaining intense secrecy around official UFO research and reports (Wendt & Duvall, 2008, p. 610).^3^ During the phase of official quiet, Wendt and Duvall suggest that the “leading role of the state distinguishes UFOs from other anomalies, scientific resistance to which is typically explained sociologically” and instead suggests a political valence given the “considerable *work* [that] goes into ignoring UFOs, constituting them as objects only of ridicule and scorn” (2008, 610). They define the result of these coordinated efforts as a UFO taboo: “a prohibition in the authoritative public sphere on taking UFOs seriously, or ‘thou shalt not try very hard to find out what UFOs are’” (Wendt & Duvall, 2008, p. 610). We might suggest that the coordinated efforts of political and scientific elites do not produce a natural silence but instead noise cancellation—the artificial absence of signal mandated by strict political and social norms.

At over a decade and a half’s distance from the publication of “Sovereignty and the UFO,” this article undertakes a mixed-method scientometric analysis of the article’s reception to assess the validity of the claim of the UFO taboo in international relations, and to better understand its operation. While bibliometric, scientometric, and citational analysis is perhaps best known in the discipline for analyses of the “gender gap” in the field (Mitchell et al, 2013; Maliniak et al, 2013; Zigerell, 2015; Dion & Mitchell, 2020; Dion et al, 2018, 2020), these techniques have been implemented in a variety of contexts to understand different kinds of trends and impacts in international relations research (Kristensen, 2015, 2018; Perrotta & Alonso, 2020; Papageorgiou & Vieira, 2021; Krymskaya, 2022). After analyzing the citation rates in the context of other work of a similar age by the authors, in the journal, and on the subject, there is no definitive proof of the existence or non-existence of a UFO taboo influencing the impact of “Sovereignty and the UFO.” However, a more in-depth analysis of citational practices *referencing* the article demonstrates how the UFO taboo operates. More than clear avoidance, belittling, or dismissing, the taboo generally continues through a tendency for references to avoid substantive engagement with the extraterrestrial aspect of the anti-anthropocentrism of the article. Rather than a taboo where people don’t talk about a subject, the UFO taboo continues because even when people *are* talking about it, they aren’t *really* talking about it. Much like Wendt’s own (1992) assessment that the institution of self-help does not follow causally from conditions of anarchy, the silence about UFOs does not follow causally from the existence of the taboo. Rather, the UFO taboo is what IR theorists make of it—and most often, it is through passive engagement.

The remainder of the paper proceeds through sections. The first provides an overview of the 2008 article, drawing particular attention to the elements of the UFO taboo described by Wendt and Duvall. The second section then turns to a discussion of the methodology that I introduce in this article as “interpretive scientometrics,” outlining the methodological precursors to this synthetic approach as well as the specific steps taken in the present investigation. The third section begins from the highest level of scientometric analysis, establishing the citational context necessary to understand the statue of “Sovereignty and the UFO” amongst its peer publications. The fourth section dives deeper into the interpretive scientometric analysis, focusing not only on *if* scholars interact with the article in question, but also *how*. This is a crucial step for understanding how the UFO taboo as an institution becomes inscribed in the fabric of the field. The final section reaffirms my argument that the UFO taboo—within the field, at least—is what IR scholars make of it and discusses what this tells us about the status of the UFO taboo argument. The conclusion also reflects on the broader applicability of interpretive scientometrics in terms of a methodology for understanding the precise processes by which knowledges are marginalized.

## “Sovereignty and the UFO” Revisited

Wendt and Duvall’s article “Sovereignty and the UFO” critiques conventional conceptualizations of sovereignty for anthropocentrism. Indeed, in setting out their argument, they claim that anthropocentrism is the key shared assumption of sovereignty debates: “Animals and Nature are assumed to lack the cognitive capacity and/or subjectivity to be sovereign; and while God might have ultimate sovereignty, even most religious fundamentalists grant that it is not exercised directly in the temporal world” (Wendt & Duvall, 2008, p. 607). If humans alone have access to sovereignty, and sovereignty is a key ordering principle of international politics, then world order relies on a deeply embedded belief in human exceptionalism. Phrased differently, the implications of the anthropocentric consensus are significant, as “anything that challenged anthropocentric sovereignty, it seems, would challenge the foundations of modern rule” (Wendt & Duvall, 2008 p. 609). Strong claims relying on a single assumption to determine their scope face a dilemma—if the assumption holds, the edifice is secure; if the assumption falls, so do the structures.

The UFO taboo becomes a taboo, then, precisely because of the importance of maintaining the current framework of sovereignty. This structural explanation holds both for academic and governmental actors; UFOs are taboo because seriously acknowledging their existence and relevance for politics would shake the anthropocentric foundation upon which all of the other conceptual and political edifices rest. At the same time, however, Wendt and Duvall note that UFOs themselves—even if not extraterrestrial—merit consideration precisely because of their conceptual significance for sovereignty in theory and practice.

It is in this context of the stakes of the sovereignty debate that Wendt and Duvall discuss the “puzzling” taboo of UFOs. They highlight the contradiction at play between the scale of evidence for UFO existence—over 100,000 sightings since 1947, many by militaries, yet an active disavowal and discrediting od UFOlogy as pseudoscience (Wendt & Duvall, 2008, p. 610). After drawing attention to the uncertainty around what UFO sightings may mean, with some less easily explained than others, they offer three key arguments for why the UFO taboo is political puzzling and scientifically problematic. I quote here at length, but reformatted:

 1. First, if any UFOs were discovered to be ETs, it would be one of the most important events in human history, making it rational to investigate even a remote possibility. It was just such reasoning that led the U.S. government to fund the Search for Extra-Terrestrial Intelligence (SETI), which looks for signs of life around distant stars. With no evidence whatsoever for such life, why not study UFOs, which are close by and leave evidence?
 2. Second, states seem eager to “securitize” all manner of threats to their societies or their rule. Securitization often enables the expansion of state power; why not then securitize UFOs, which offer unprecedented possibilities in this respect?
 3. And finally, there is simple scientific curiosity: why not study UFOs, just like human beings study everything else? At least something interesting might be learned about Nature. Notwithstanding these compelling reasons to identify UFOs, however, modern authorities have not seriously tried to do so. This suggests that UFO ignorance is not simply a gap in our knowledge, like the cure for cancer, but something actively reproduced by taboo. (Wendt & Duvall, 2008, p. 611).

Following any one of these threads should provide a *prima facie* case for scientific study, and without evidence that would concretely deny the possibility of the extraterrestrial hypothesis, Wendt and Duvall conclude that (2008, 613-618) scientific reasoning cannot explain the UFO taboo. Instead, they argue, the choice of ignorance is impelled by the anthropocentric metaphysics of sovereignty.

While far from a *probing* review of the article, I hope that this context for the specific discussion around the UFO taboo provides sufficient context to assess what we should expect from “the literature” in its treatment of “Sovereignty and the UFO.” To begin with a negation, we should not expect an evidence-based refutation of the argument. Instead, the institutional marginalization of the topic and discrediting of the logic of the analysis would function to disempower the potentially extraterrestrial critique of anthropocentrism. At the distance of a decade and a half, the article has received tens of thousands of downloads, and was among the top-10 most read from October 2022 to March 2023.^4^ As one reviewer noted, spikes in interest occurred following UFOlogical milestones in the United States, such as the publication of the 2021 Pentagon Report—which famously identified unidentified aerial phenomena as a potential threat while also highlighting that “sociocultural stigmas” join technical limitations as “obstacles to collecting data on UAP” (Office of the Director of National Intelligence, 2021, p. 4)—and following more recent American legislation that established the All-Domain Anomaly Resolution Office and formalized reporting processes for unidentified aerial phenomena.^5^ Even still, this overall download figure does not provide much information. Thankfully, while downloads rates have little depth; citations tell more of a story.

## Methodology

To better assess the stickiness and operation of the UFO taboo, this article adopts a scientometric methodology. Along with the related fields of bibliometrics and informetrics, scientometrics has long been dominated by quantitative and statistical methodologies (Hood & Wilson, 2001). Often conflated, I follow the distinction between the fields offered by Jean Tague-Sutcliffe (1992, p. 1), which understands bibliometrics to refer to the study of “the production, dissemination, and use of recorded information,” scientometrics to refer to the study of “science as a discipline or economic activity” and “part of the sociology of science,”^6^ and informetrics as the broader study of “information in any form, not just records or bibliographies, and in any social group, not just scientists.” Despite the overwhelming advantage of quantitative methodologies in the volume of scholarship published in the field, an often-overlooked tradition of “qualitative scientometrics” (Callon et al., 1986) has advanced important insights about the scientific practices that lead to broader trends that form the basis of quantitative scientometric analysis (e.g., Leydesdorff, 1989; Zavaraqi & Fadaie, 2012; Mingers & Leydesdorff, 2015). Often, this qualitative work draws on sociological insights from the same corners of science and technology studies (e.g., Latour & Woolgar, 1986; Latour, 1987; Barad, 2007) which have recently informed methods debates in critical international relations. Qualitative scientometrics are valuable because we know so little about how seriously citations engage with sources in a general sense versus how often sources appear as a chain of “passing references and obligatory footnotes” (Walker, 1993, p. 29)—this important distinction is invisible from the vantage point of top-line totals. This broader, multi-method approach to scientometrics guides the methodological design of the present investigation. I seek to first identify and then understand the disciplining impact of the UFO taboo, taking the network of articles surrounding Wendt and Duvall (2008) as the empirical basis for understanding disciplinary practices and the sociology of international relations research.

Understood through the disciplinary lens of international relations theory, I describe this research design as “interpretive scientometrics.” Interpretive methods are often situation-specific, focusing on language and sense-making (Yanow, 2007), and in a particular way drawing attention to the ways in which power and social interactions shape the meaning of human experiences (Lynch, 2013). In terms of their disciplinary sociology, interpretive methods understand “scientific work as a practice…that seeks to persuade others of the ‘goodness’ of its findings,” (Schwartz-Shea & Yanow, 2013, p. 3) and in so doing recognize the sociality of social science research, replete with power relations sometimes more readily identified by scholars than among scholars. While often qualitative and phenomenological in methods, interpretive scholarship can also employ quantitative approaches to knowledge-production (Sjoberg & Horowitz, 2013; Barkin & Sjoberg, 2015, 2017). In applying both quantitative and qualitative techniques for collecting and analyzing data, this study brings together mixed-method approaches from two unbalanced fields—the quantitatively dominated field of scientometrics and the qualitatively dominated interpretive social research—to consider a specific dataset: the citation web of “Sovereignty and the UFO.”^7^

Data collection began with a search of citations for “Sovereignty and the UFO” on Google Scholar. While Google Scholar has been criticized for metadata errors, its inclusive search criteria compared to other options means that its citation data captures a more diverse and global sample than other aggregators such as Web of Science (e.g, Harzing & Alakangas, 2016; Lohaus et al, 2021). Because both Wendt and Duvall have active Google Scholar profiles, their personal pages were then examined for temporally adjacent works to capture citation data of comparably aged texts. The GS citation rates for all research articles (not book reviews or editorial items) from the 2008 volume of *Political Theory* were catalogued. The final comparative analysis included a manual review of all journal volumes from 2007, 2008, and 2009 from a variety of journals generally regarded as high-impact venues for international relations research.^8^ All research articles (not book reviews or editorial items) with “Sovereignty” in the title were collected and GS citation rates were collected. To ensure consistency and comparability, all GS citation data were collected within the same refresh cycle (Murphy et al, 2023, p. 5).^9^

To analyze the citations *of* “Sovereignty and the UFO,” citing works were limited to journal articles published in English. The rationale for the genre restriction is that in the field of international relations, “journals are the most direct measure of the discipline itself” (Waever, 1998, p. 697). While recognizing the deleterious impacts of the anglonormativity of international relations (D’Aoust, 2012; Grondin et al, 2012; Grondin, 2014; Murphy & Wigginton, 2020), the decision to opt for English-language publications only was based on researcher competence in the languages of works citing “Sovereignty and the UFO.”^10^ All of the articles meeting these selection criteria were manually reviewed by the author. Citation data for the articles citing Wendt & Duvall (2008) were recorded from Google Scholar during the same refresh cycle as noted above. Journal ranking data were drawn from SCImago Journal Rankings.^11^ This was chosen after an initial review of journal Impact Factors revealed that not all journals were ranked. While the IF provides a more precise measurement for citation-based ranking, connection to the dataset was prioritized over precision in ranking high-impact journals. Interpretive data relating to the type of discussion was coded as either “passing” or “substantive” depending on the level of detail with which a citing article engaged with the substance of “Sovereignty and the UFO”—passing references were understood as single parenthetical references without specific discussion, single-sentence literature review references, and footnote-only engagement. Works were also described based on their use or non-use of the term “UFO,” “unidentified flying object,” or “extraterrestrial,” to test if the taboo applied to the term itself or to the broader concept.

## Comparative Context

Citations are the currency of scholarly impact within the academic community. Within international relations theory, the role of references in the construction of the discipline is well-established (Walker, 1993, 29). Establishing the citational context of a work helps to better the scope of a work’s reception vis-à-vis its peers, which can be defined in multiple ways. This contextual piece is therefore less directly focused on the specific definition of the UFO taboo defined by Wendt and Duvall, and instead starts at the broadest part of the funnel, asking if the authors’ piece on UFOs has been ignored in absolute quantitative terms relative to peer scholarship. With context in hand, we can better appreciate the scholarly stature of “Sovereignty and the UFO” as a scientific object. To better understand the reception of the 2008 article in citational terms, it is important to first place the work in the context of the author’s own bodies of work. Both Alexander Wendt and Raymond Duvall have had influential careers in the field, so to compare their article against the average citation rate of the field *writ large* would risk underestimating their place in the field. Therefore, the first comparative analysis is to compare “Sovereignty and the UFO” with other published work by the two others within a similar timeframe. Table 1 presents the UFO article in the context of Wendt’s three previously and subsequently published articles.

**Table 1.** “Sovereignty and the UFO” in the context of Wendt’s published work.

1307	Wendt, Alexander. “Why a world state is inevitable.” *European journal of international relations* 9, no. 4 (2003): 491-542.
857	Wendt, Alexander. “The state as person in international theory.” *Review of international Studies* 30, no. 2 (2004): 289-316.
441	Wendt, Alexander. “Driving with the rearview mirror: On the rational science of institutional design.” *International Organization* 55, no. 4 (2001): 1019-1049.
125	Wendt, Alexander, and Raymond Duvall. “Sovereignty and the UFO.” *Political Theory* 36, no. 4 (2008): 607-633.
55	Snidal, Duncan, and Alexander Wendt. “Why there is International Theory now.” *International Theory* 1, no. 1 (2009): 1-14.
36	Der Derian, James, and Alexander Wendt. “‘Quantizing international relations’: The case for quantum approaches to international theory and security practice.” *Security Dialogue* 51, no. 5 (2020): 399-413.
14	Wendt, Alexander. “The mind–body problem and social science: Motivating a quantum social theory.” *Journal for the Theory of Social Behaviour* 48, no. 2 (2018): 188-204.

“Sovereignty and the UFO” falls neatly in the middle of Wendt’s work from this time, with a lower number of citations than the three articles preceding it but a higher number than the three that would follow. As the median article during this period, it is difficult to conclude that the citational impact is deflated due to the taboo. In the case of Raymond Duvall’s work in the period displayed in Table 2, “Sovereignty and the UFO” in fact ranks second behind only Barnett & Duvall, (2005). Therefore, the evidence of a taboo depressing impact is even less than in the case of Wendt. Yes, the citation rate is lower than other works published by the authors, but that does not mean that an exogenous taboo is operating.

**Table 2.** “Sovereignty and the UFO” in the context of Duvall’s published work.

2549	Barnett, Michael, and Raymond Duvall. “Power in international politics.” *International organization* 59, no. 1 (2005): 39-75.
125	Wendt, Alexander, and Raymond Duvall. “Sovereignty and the UFO.” *Political Theory* 36, no. 4 (2008): 607-633.
52	Duvall, Raymond, and Latha Varadarajan. “On the practical significance of critical international relations theory.” *Asian Journal of Political Science* 11, no. 2 (2003): 75-88.
33	Duvall, Raymond, and Latha Varadarajan. “Traveling in paradox: Edward said and critical international relations.” *Millennium* 36, no. 1 (2007): 83-99.
32	Duvall, Raymond, and Jonathan Havercroft. “Taking sovereignty out of this world: space weapons and empire of the future.” *Review of International Studies* 34, no. 4 (2008): 755-775.
29	Chowdhury, Arjun, and Raymond Duvall. “Sovereignty and sovereign power.” *International Theory* 6, no. 2 (2014): 191-223.
16	Havercroft, Jonathan, and Raymond Duvall. “Challenges of an agonistic constructivism for international relations.” *Polity* 49, no. 1 (2017): 156-164.

While we might conclude “no taboo” given the middle-to-upper impact of the paper in terms of the authors’ work of a similar age, one might respond that the journal where an article is published could have an influence on the citation rate of published works. After all, while journal ranking metrics differ in their precise methodology, they share a primary assumption that there is a connection between journal quality and citation rates (Falagas et al, 2008; Delgado-Lopez-Cozar & Cabezas-Clavijo, 2013). If the journal’s past prestige influences citations of future works, then it is important to compare articles with their in-journal peers when assessing relative impact.

“Sovereignty and the UFO” appeared in the journal *Political Theory,* a journal that offers a platform for theoretical debates in its namesake subfield but also innovative theoretical interventions from other perspectives, including international relations. To provide a fair comparison within the context of this journal, Table 3 presents the citation rates of all articles published in volume 36 of *Political Theory* (where “Sovereignty and the UFO” appeared). As with the authorial context comparisons, we again find that the article falls in the middle-to-upper tier in terms of citations. While all articles from the volume received citations, many received far fewer than “Sovereignty and the UFO,” and many fewer received more. In the context of the journal’s citation, it is difficult to identify this single article as one impacted by a taboo given its placement in terms of citation.

**Table 3.** “Sovereignty and the UFO” in the context of Political Theory, vol. 36.

844	Abizadeh, Arash. “Democratic Theory and Border Coercion: No Right to Unilaterally Control Your Own Borders.” *Political Theory* 36, no. 1 (2008): 37-65.
203	Markell, Patchen. “The insufficiency of non-domination.” *Political theory* 36, no. 1 (2008): 9-36.
155	Farr, James. “Locke, natural law, and new world slavery.” *Political Theory* 36, no. 4 (2008): 495-522.
143	Cohen, Jean L. “Rethinking human rights, democracy, and sovereignty in the age of globalization.” *Political theory* 36, no. 4 (2008): 578-606.
125	Wendt, Alexander, and Raymond Duvall. “Sovereignty and the UFO.” *Political Theory* 36, no. 4 (2008): 607-633.
125	Luxon, Nancy. “Ethics and subjectivity: Practices of self-governance in the late lectures of Michel Foucault.” *Political Theory* 36, no. 3 (2008): 377-402.
102	Muthu, Sankar. “Adam Smith's critique of international trading companies: theorizing ‘globalization’ in the Age of Enlightenment.” *Political Theory* 36, no. 2 (2008): 185-212.
97	Nelson, Eric. "From primary goods to capabilities: distributive justice and the problem of neutrality." *Political theory* 36, no. 1 (2008): 93-122.
63	De Roover, Jakob, and S. N. Balagangadhara. “John Locke, Christian liberty, and the predicament of liberal toleration.” *Political Theory* 36, no. 4 (2008): 523-549.
60	Smith, Rogers M. “Religious rhetoric and the ethics of public discourse: the case of George W. Bush.” *Political Theory* 36, no. 2 (2008): 272-300.
54	Schwartzberg, Melissa. “Voting the general will: Rousseau on decision rules.” *Political theory* 36, no. 3 (2008): 403-423.
49	Vatter, Miguel. “The idea of public reason and the reason of state: Schmitt and Rawls on the political.” *Political Theory* 36, no. 2 (2008): 239-271.
38	Sreedhar, Susanne. “Defending the Hobbesian right of self-defense.” *Political Theory* 36, no. 6 (2008): 781-802.
34	Breiner, Peter. “Machiavelli's ‘new prince’ and the Primordial Moment of Acquisition.” *Political Theory* 36, no. 1 (2008): 66-92.
33	Baumgold, Deborah. “The difficulties of Hobbes interpretation.” *Political theory* 36, no. 6 (2008): 827-855.
32	Feldman, Leonard C. “Judging necessity: Democracy and extra-legalism.” *Political Theory* 36, no. 4 (2008): 550-577.
27	Leeb, Claudia. “Toward a theoretical outline of the subject: the centrality of Adorno and Lacan for Feminist political theorizing.” *Political Theory* 36, no. 3 (2008): 351-376.
25	Booth, W. James. “The color of memory: Reading race with ralph ellison.” *Political Theory* 36, no. 5 (2008): 683-707.
22	Patapan, Haig, and Jeffrey Sikkenga. “Love and the Leviathan: Thomas Hobbes's Critique of Platonic Eros.” *Political Theory* 36, no. 6 (2008): 803-826.
21	Shulman, George. “Thinking Authority Democratically: Prophetic Practices, White Supremacy, and Democratic Politics.” *Political Theory* 36, no. 5 (2008): 708-734.
20	Turner, Jack. “Awakening to race: Ralph Ellison and democratic individuality.” *Political Theory* 36, no. 5 (2008): 655-682.
20	Ferguson, Kathy E. “Discourses of danger: locating Emma Goldman.” *Political theory* 36, no. 5 (2008): 735-761.
15	Vetter, Lisa Pace. "Harriet Martineau on the theory and practice of democracy in America." *Political Theory* 36, no. 3 (2008): 424-455.
14	Miller, Ted H. “The Two Deaths of Lady Macduff: Antimetaphysics, Violence, and William Davenant's Restoration Revision of Macbeth.” *Political Theory* 36, no. 6 (2008): 856-882.
6	Jenco, Leigh K. “Theorists and Actors: Zhang Shizhao on ‘Self-Awareness’ as Political Action.” *Political theory* 36, no. 2 (2008): 213-238.

An objection to the in-journal comparison, however, may be that the multi-subfield nature of *Political Theory* would compare “Sovereignty and the UFO” to articles not connected to international relations theory. This objection merits consideration because of the differential citation rates observed between subfields (Montpetit et al, 2008)—at least in the case of the journal comparison, if not the authorial comparisons. To address this, a search for articles discussing “sovereignty” in leading journals of international relations^12^ was performed to establish a comparator pool for “Sovereignty and the UFO,” from the period of 2007–2009. Table 4 again presents the findings rank-ordered by citation count. As can be observed, we again find “Sovereignty and the UFO” in the middle-to-upper tier of articles by citation, a mere two citations from the pole position among 2008 articles. This thematic comparison demonstrates that the article in question has been cited at a higher rate than other works on a similar topic of a comparable vintage.

**Table 4.** “Sovereignty and the UFO” in the context of articles with “Sovereignty” in the title (2007–9).

251	Benhabib, Seyla. “Claiming rights across borders: International human rights and democratic sovereignty.” *American Political Science Review* 103, no. 4 (2009): 691-704.
171	Vaughan-Williams, Nick. “The generalised bio-political border? Re-conceptualising the limits of sovereign power.” *Review of International Studies* 35, no. 4 (2009): 729-749.
127	Johnson, Carter. “Partitioning to peace: Sovereignty, demography, and ethnic civil wars.” *International Security* 32, no. 4 (2008): 140-170.
125	Wendt, Alexander, and Raymond Duvall. “Sovereignty and the UFO.” *Political Theory* 36, no. 4 (2008): 607-633.
98	Suganami, Hidemi. “Understanding sovereignty through Kelsen/Schmitt.” *Review of International Studies* 33, no. 3 (2007): 511-530.
70	Pratt, Nicola. “The Queen Boat case in Egypt: sexuality, national security and state sovereignty.” *Review of International Studies* 33, no. 1 (2007): 129-144.
44	Zevnik, Andreja. “Sovereign-less Subject and the Possibility of Resistance.” *Millennium* 38, no. 1 (2009): 83-106.
38	Evans, Mark. “Balancing Peace, Justice and Sovereignty in Jus Post Bellum: The Case of Just Occupation'.” *Millennium* 36, no. 3 (2008): 533-554.
35	Kayaoglu, Turan. “The Extension of Westphalian Sovereignty: State Building and the Abolition of Extraterritoriality.” *International Studies Quarterly* 51, no. 3 (2007): 649-675.
32	Duvall, Raymond, and Jonathan Havercroft. “Taking sovereignty out of this world: space weapons and empire of the future.” *Review of International Studies* 34, no. 4 (2008): 755-775.
24	Karp, David Jason. “The utopia and reality of sovereignty: social reality, normative IR and ‘Organized Hypocrisy’.” *Review of International Studies* 34, no. 2 (2008): 313-335.

Each of the four comparative contexts have unique strengths and weaknesses for analysing the stickiness of the UFO taboo to the article “Sovereignty and the UFO.” Authorial comparisons may calibrate an article’s citational impact to the individuals’ status in the field, but miss out on broader trends. Journal-based comparisons may adjust for venue prestige with the trade-off of effacing inter-field citation dynamics. Thematic comparisons may provide a benchmark for interventions on a given topic, while also providing a limited insight into the individuals’ influence within a field. The important insight from the analyses presented in this section discussing the context of “Sovereignty and the UFO” is not the findings of one or another table. Rather, the significance follows from the similarity across comparative methods. The authorial comparisons demonstrate that while the article is not the highest-cited work by either Wendt or Duval during the period, it is also not the least-cited. This means that when read with an eye to the potential influence of an author on impact, the paper has been reasonably well-received. The story is largely the same when comparing “Sovereignty and the UFO” to the remainder of the volume of *Political Theory*; the article is not the leader, but sits comfortable in the middle-to-upper tier of scholarship published in the group.

Given the broad consensus on the echelon of the article’s impact when examined vis-à-vis peers by multiple definitions, it does not appear to be a feasible claim that a UFO taboo necessarily causes the ignorance of the topic of UFOs within the field of international relations. But what if there is a more specific mechanism at work—not a blanket taboo that pre-exists the practice of scholarship as a condition of the field, but instead a softer kind of taboo that emerges through the references to work on the UFO? Inspired by Wendt’s own argument about self-help under conditions of anarchy in world politics, I would like to suggest that the institution of the UFO taboo does not necessarily follow from the anarchy of scholarly practice but is instead constructed through the (non-)engagement with the article.

## Follow the Citations!

To understand the practice and operation of the UFO taboo, we must—borrowing an interpretive page from the book(s) of actor-network theory—follow the citations. Bruno Latour draws attention to the practice of citation in *Science in Action*, saying that the referenced article “is not only referred to; it is also qualified…instead of passively linking their fate to other papers, the article *actively* modifies the status of these papers” (1987, 35). In so doing, the article doing the referencing produces a “context of citation” that “shows us how one text acts on others to make them more in keeping with its claims” (Latour, 1987, 35). Citations are significant and active elements of the cycles of credit that construct knowledge claims (Latour & Woolgar, 1986), through processes of translation, problematization, and association (Callon et al, 1986). By *following the citations themselves*,^13^ we will see not only the overall quantitative expression of the citational impact of “Sovereignty and the UFO” but a further degree of the meaning within those citations. This interpretive approach to scientometrics offers a greater appreciation of the meaning and agency bound up in citational practice. As noted in the methodology section, this section refers to English-language journal articles only in an attempt to capture the hegemonic “center” of the field of international relations.

**Figure 1.**
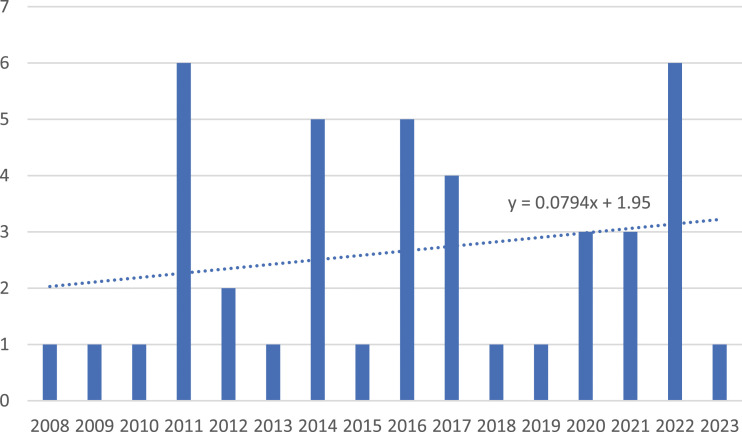
Citations of “Sovereignty and the UFO” over time.

Figure 1 offers an overview of the temporal dimension of citation related to “Sovereignty and the UFO.” It is typical for citations to lag publication given the timelines of scholarly publishing, and while single citations exist in 2008, 2009, and 2010, larger numbers begin to appear in 2011. While there is, overall, a slightly upward trend in citation rate, the trendline demonstrates the gentleness of this rise. It remains to be seen whether the release of UFO-related documents since 2019 will lead to a growth in the citation rate, but at the very least it is clear that institutional acknowledgement of UFOs was not required to provide “cover” for the citation of “Sovereignty and the UFO” in published research.

While the measurement of quality of an article is a contested process, two proxy measures can offer a window into the implications of the prestige economy of academic publishing at play in the articles citing “Sovereignty and the UFO.” To this end, Table 5 sorts all citing articles by their SCImago journal ranking quartiles, while Table 6 provides high-level citation data for the citing articles. While not all articles are published in top-tier journals, this would hardly be a reasonable expectation for a selection of articles, and it is worth noting that the distribution of articles skews to first quartile journals overall. Similarly, while some articles have never been cited according to Google Scholar metrics, many have been cited at very respectable rates, including Lake (2011) over 400 times and Vucetic (2011) over 100 times. Considering the field as a whole, it would seem as though the potential taboo-contagion that may be expected to follow from articles citing “Sovereignty and the UFO” does not occur.

**Table 5.** Articles citing “Sovereignty and the UFO” by SJR quartile.

Q1	22
Q2	3
Q3	6
Q4	4
NR	7

**Table 6.** Citation metrics of articles citing “Sovereignty and the UFO.”

Avg (SD)	24.5 (64.5)
0 cite	9
>10	16
>50	4
>100	2

One may wonder the specificity of the taboo, however, which would imply that the specific invocation of the letters “UFO” may be more hotly contested than euphemistic or indirect references. Tables 7 and 8 disaggregate the journal quartile and citation metrics to isolate articles that explicitly mention “UFOs” from those that do not use the term. In both cases, the differences largely wash out, especially considering the differences in size. A higher percentage of citing articles not using the term UFO appear in both Q1 and unranked journals, and while there is a higher average citation, the outlier of Lake (2011) renders the average almost meaningless. Citing articles explicitly mentioning UFOs have a flatter distribution across journal quartiles, but a higher number hitting citation benchmarks of 10, 50, and 100. Ultimately, it seems as though the “UFO taboo” is not influenced in a meaningful way by the specific use of non-use of the term “UFO.”

**Table 7.** SJR quartile disaggregated by use/non-use of “UFO” in article text.

	No UFO	UFO
Q1	9 (64%)	13 (46%)
Q2	0 (0%)	3 (11%)
Q3	1 (7%)	5 (18%)
Q4	0 (0%)	4 (14%)
NR	4 (29%)	3 (11%)

**Table 8.** Citation metrics disaggregated by use/non-use of “UFO” in article text.

	No UFO	UFO
Avg (SD)	39.8 (108.3)	16.9 (26.2)
0 cite	4 (29%)	5 (17%)
>10	6 (43%)	10 (36%)
>50	1 (7%)	3 (11%)
>100	1 (7%)	1 (4%)

Such high-level summaries do not offer much insight into what the actor-network theorists call the translation, association, and problematization bound up in the practice of academic citation. After all, there are many different ways in which texts are referenced in the context of academic articles. These qualitative differences in citation are material to the understanding of the impact of “Sovereignty and the UFO” because the conditions of discussion offer a more meaningful account than the broader summaries expressed above in quantitative terms. The remainder of the discussion is more concerned about *how* authors interact with the content of the argument about UFOs and UFOlogy than *if* the articles employ the term or cite “Sovereignty and the UFO.” I continue to reflect on the entire pool of English-language articles, but divide them into “passing” and “substantive” categories as noted below.

A review of the citing articles revealed a notable difference between passing references and substantive discussions of “Sovereignty and the UFO.” Articles that included a footnote or parenthetical reference either as a function of general signaling or nonspecific acknowledgement that conceptual debates around sovereignty exist were coded as “passing,” while engagements of a sentence or more with the content of the argument were coded as “substantive.” Samples of passing references are offered below:

 • “Positing the existence of and generating knowledge about something that we cannot yet experience, perhaps because the specialized equipment needed to perceive and measure it has yet to be constructed (Harré, 1985:93–95), poses no special problems for the pragmatist emphasis on experience; problems are, however, posed when we start claiming to have knowledge of quarks, higher dimensions, or social structures understood as deep generative potentialities (Wendt, 1987; Wendt and Duvall, 2008)” (Jackson, 2009, 656).
 • “International studies deals with the largest and most complicated social system possible (barring extraterrestrials, see Wendt and Duvall, 2008; or perhaps zombies, see Drezner, 2011)” (Lake, 2011, 467).
 • “The concept of state sovereignty traces back to the Peace of Westphalia, but, despite a fairly clear origin, the idea has multiple meanings (Joffe, 1999; Wendt and Duvall, 2008)” (Mobley, 2016, 130).
 • “This article focuses on relations constituting the domain of IR. The international conceptually includes relations of nations of beings (human and non-human alike). Its reach is both cosmic and microcosmic so it can range from intergalactic relations to the nations of microbial beings that constitute the foundation of much of the planet. See Alexander Wendt and Raymond Duvall, ‘Sovereignty and the UFO’…” (Trownsell, 2022, 801n1).

While Lake (2011) and Trownsell (2022) gesture towards the consideration of extraterrestrials, this is done in passing and does not substantially inform the line of argumentation. In other circumstances, such as Jackson (2009) and Mobley (2016), we find that the UFO context of Wendt and Duvall’s argument is removed from the broader point about historical factors and anthropocentric sovereignty.

Substantive engagements with “Sovereignty and the UFO” took varying forms. In some cases, the article is taken as a phenomenon, grounding a discussion of the UFO taboo or the limits of international relations scholarship:

 • “Several years ago, Alexander Wendt and Raymond Duvall wrote a piece entitled ‘Sovereignty and the UFO’ in which they reflected critically on the unwillingness of governments (and by extension, security studies scholars) to attend to the possibility of first contact—possibly hostile first contact—from beyond our biosphere (Wendt and Duvall, 2008). The article was not widely cited and was seen by some as pushing the boundaries of acceptable thinking in security studies...” (Carpenter, 2016, 94).
 • “If the simulation is to function logically, the logic of levels and in regularly asserting IR’s stature at the highest level implies that future IR work would need to move up yet another level, to interstellar relations. This logic of moving up the levels is evident in Alexander Wendt and Raymond Duvall’s examination of Unidentified Flying Objects (UFO) and the anthropocentricity of sovereignty, although they are not the first social scientists to think about this final frontier” (Baron, 2015, 262).

In other occasions, the conceptual argument about how UFOs challenge anthropocentric sovereignty is brought into conversation with other theoretical debates in international relations, such as security communities and territoriality:

 • “Similarly, Wendt & Duvall (2008) argue that the possibility that unidentified flying objects (UFOs) indicate the existence of alien life forms in our solar system is repressed in contemporary public discourse. In their account, the state’s authority is threatened by the possibility of non-human life forms powerful enough to reach Earth, because it calls into question the human status as sovereign and the state’s status as provider of physical and ontological security…” (Mitzen, 2016, 237-8).
 • “Material and ideational/ontological threats overlap, and many of the threats addressed in our cases exhibit aspects of both. A parallel argument is presented by Wendt and Duvall (2008, pp. 620–622), who suggest that the potential existence of extraterrestrial life, in the form of UFOs, threatens the state both materially and ontologically, resulting in a ‘UFO taboo’ in which UFOs are effectively ignored by authorities” (McKay et al, 2014, 117n11).

It is notable that while some substantive engagements have explicitly focused on extraterrestrials (e.g., Harrison, 2011; Eghigian, 2017), the examples highlighted above have not. For non-UFOlogical references to “Sovereignty and the UFO,” perhaps the most remarkable observation is that the article is often treated as *unremarkable*. Occasionally there is a reference to a chilly reception (Carpenter, 2016), but more often the article is cited as if any other intervention in the theoretical debates on sovereignty—with UFOs as a case study functionally equivalent to any other. At times, this may appear as a gentle endorsement of the UFO taboo by acknowledging its existence; however, rendering the UFO taboo as a quotidian gap removes the exceptionality intended in “Sovereignty and the UFO,” which posited something special about the relationship between the UFO taboo in particular and the anthropocentric foundations of sovereignty. In making the acceptance of a “UFO taboo” palatable as a matter of course, the article can be summarized as presenting a fact of the field rather than outlining a call to action.

As Table 9 demonstrates, while both passing and substantive engagements included mostly neutral framings of the article, substantive engagements were more likely to be positive. What stands out further from the table, however, is the nearly 2:1 ratio by which scholars favor passing mentions over substantive engagements. This distinction between passing reference and substantive engagement is theoretically significant, as one entails—to return to the language of the actor-network theorists—a close association of the *citing* work with the *cited* work. The substantive engagement need not be positive, and problematizations are offered as a key function of citation for precisely this reason. The prevalence of passing references means that the associations are weaker rather than stronger, and that the citing authors end up with a less proximate connection to UFOlogy.

**Table 9.** Framing of passive and substantive engagements.

	Passing	Substantive
Positive	3 (11%)	4 (27%)
Negative	1 (4%)	0 (0%)
Neutral	23 (85%)	11 (73%)
TOTAL	27	15

**Figure 2.**
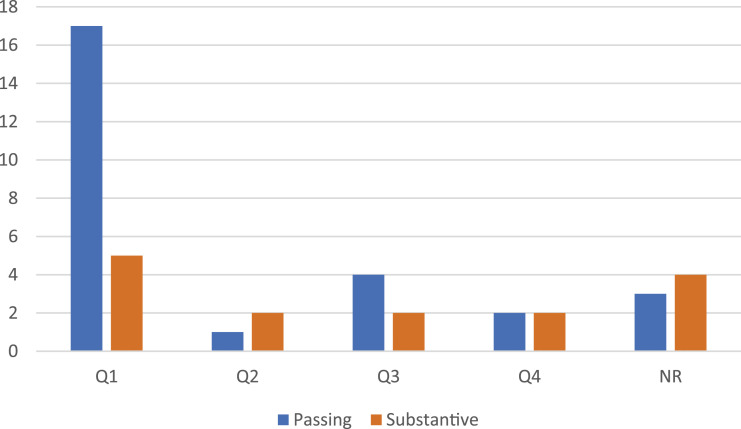
Journal ranking by passive and substantive engagements.

While the use or non-use of the term “UFO” did not have a material impact on the prestige economy of the journal, the strategies of association implied by passing or substantive references to “Sovereignty and the UFO” accompany differential outcomes in terms of journal ranking and article citation. Figure 2 displays the quartile journal ranking of venues publishing articles that employ weaker and stronger associative strategies with regards to “Sovereignty and the UFO.” Citing articles in Q1 journals are much more likely to employ a weaker associative strategy than a strong one, with seventeen passive reference articles compared to five engaging substantively. Not only is this a substantial increase over the general ratio of 2:1, but it also represents the entire “advantage” of passive over substantive reference. Despite the overall ratio of 2:1, the absolute number of articles appearing in lower-tier and unranked journals is equal between passing references and substantive engagements. Whether authorial intent or products of the review process, when articles in higher-ranking journals engage with “Sovereignty and the UFO,” the content of the article is treated less substantively such that the citing article (and journal) are less closely associated with Wendt and Duvall’s specific discussion of UFOlogy.

But a difference between passing and substantive engagement is not only visible in the placement of the citing articles; the citations received by these texts reveal a continuation of the pattern. Table 10 provides the same citation metrics as noted above, disaggregated this time in terms of their strategy of association. While the ratio of 2:1 holds steady for 0-cite articles, all of the higher benchmarks tilt in favor of articles that include only a passing reference to “Sovereignty and the UFO.” This is notable that no article engaging substantively with UFOlogy and/or extraterrestrials has reached the 100-citation threshold.

**Table 10.** Citation metrics by passive and substantive engagements.

	Passing	Substantive
Avg (SD)	31.4 (80.3)	12.1(18.0)
0 cite	6 (22%)	3 (20%)
>10	11 (41%)	5 (33%)
>50	3 (11%)	1 (7%)
>100	2 (7%)	0 (0%)

As an interpretive approach to scientometrics, my aim in this article is not to infer causal mechanisms but to understand the meaning that different strategies of association produce in the context of citational practices. In the case of “Sovereignty and the UFO,” we find that articles appearing in more prestigious journals and receiving high rates of citations are likely to incorporate strategies of weak association with “Sovereignty and the UFO” (if they cite the work at all). Rather than a dense web of citational connections that substantively engage with the unique UFOlogical and extraterrestrial discussions of the work, we instead find that passing references communicate a reluctance to associate with the content of the text. The UFO taboo operative in the context of “Sovereignty and the UFO” is not one that ostracizes the text or marks its citers as unacceptable for publication. Rather, in the practice of citing the article, the UFO taboo is reconstructed—complete with some exceptions to prove the rule.

## Conclusion and Discussion

This article has offered an exploration of the reception of Wendt and Duvall’s “Sovereignty and the UFO,” to explore their core argument about the existence of a UFO taboo in international relations theory. Through an innovative methodological design of interpretive scientometrics, the citational analysis undertaken in this article investigated the meaning-making practices embedded in the knowledge-production processes through citation. In recognizing the slight tension between quantitative and qualitative aspects of interpretive scientometrics, this methodology helps to refine our understanding of the status of the UFO taboo: there is a narrow taboo on the discussion of UFOs, but there is no “UFO taboo” taboo. Citers have intersubjectively reconstructed the meaning of the parenthetical “Wendt & Duvall 2008” to refer less directly to the specific taboo against UFOlogy and more generally towards a critique of anthropocentric sovereignty and the “UFO taboo” as a reality of scholarly practice—a complexity difficult to appreciate with traditional scientometrics alone. This conclusion expands on two points: the UFO taboo/“UFO taboo” taboo differentiation as an episode of meaning construction in scholarly practice, and what this case study tells us about the broader applicability of interpretive scientometrics for understanding International Relations as a field of scholarly practice.

In their article, Wendt and Duvall (2008, 610-611) describe the UFO taboo as an active of discrediting UFOlogy as pseudoscience, a prohibition on public discussion, and a reproduced ignorance. From the interpretive scientometric analysis of “Sovereignty and the UFO,” I would like to suggest that while there is some evidence to support the identification of a UFO taboo, it is not nearly as emphatic or definitive in its impact. First, there is little evidence that discussion of the article has been prohibited. When compared to peer works defined in various forms, the article continually appears in the middle-to-upper tier of citation rates. Furthermore, the text continues to receive a large volume of downloads from the website of *Political Theory*, long after its publication. Second, there is little evidence to support that active discrediting is underway (at least in the published literature). The bulk of the literature citing “Sovereignty and the UFO” is either neutral or positive in its engagement. However, there is one important way in which the citational data reveal an important activity of taboo-construction: through a passive reference to the text. It is not that the UFO prevents people from talking about UFOs, but that it prevents people from *really* talking about UFOs. To this end, while we might agree with Wendt and Duvall that there is a taboo against explicit UFOlogy in International Relations theory, reference to the taboo or the article discussing it is not in itself taboo—there is no “UFO taboo” taboo. Indeed, interaction patterns with the paper have tended to either note the existence of the taboo as a matter of fact *or* to present the paper as an argument primarily about anthropocentrism rather than UFOlogy. In the time since the article was published, a variety of approaches have forwarded non-anthropocentric ontologies drawing on theories of interspecies and microbial relations, new materialism, and beyond (Youatt, 2014; 2023; Voelkner, 2019; 2021; 2022; Salter, 2015; 2016).

My suggestion at this juncture is that the UFO taboo is what IR theorists make of it. This was not a necessary outcome of the citational ecosystem that developed around “Sovereignty and the UFO,” as we could have imagined multiple ways in which scholars might have engaged with the piece—true ignorance, skeptic debunking, or UFOlogical engagement. Scholarly taboos are not fixed and necessary imperatives in the prestige economy of academic publishing, but are instead socially constructed phenomena that are continually (re)produced through the strategies of association that scholars employ in their citation and non-citation of texts. The UFO taboo example, then, is not only a case that helps us to understand the status of extraterrestrials in challenging the anthropocentrism of sovereignty, but also many other norms in scholarly discourse that are reproduced through citational practice.^14^ Rather than merely a structural category, taboos in scholarly discourse are also mediated by the intersubjective creation of meaning—just as the interaction of states could have created institutions other than self-help, other strategies of scholarly engagement with “Sovereignty and the UFO” might have broadened or undone the UFO taboo instead of the narrowing that we have witnessed. This recognition of scholarly agency in the construction of meaning is important because it reveals how interpretive scientometrics might be put to work in broader contexts within the field.

After all, absences and silences are commonplace in International Relations, and marginalization of subjects and perspectives too often limit the fuller appreciation of our field. For many decades, gender was ignored by the field of international relations, justified not on the grounds of an explicit taboo but on claims that the focus on power politics did not require attention to the personal and private (cf. Enloe, 1989). Women-identifying authors were also omitted from the canon (Owens, 2018; Hutchings & Owen, 2021), left off of course reading lists (Colgan, 2017; Phull et al, 2019), and undercited in journals (Mitchell et al, 2013; Maliniak et al, 2013; Zigerell, 2015; Dion & Mitchell 2019; Dion et al, 2018; 2020). Similar phenomena have been observed in the marginalization of non-Western, racialized, queer, Indigenous, and other perspectives and subject areas (Andrews, 2022; Sondarjee, 2023). Interpretive scientometrics can offer a new toolkit for the analysis of these and other absences and silences within the published discipline of international relations. As calls for a more inclusive and global discipline abound, it is more important than ever to develop and deploy robust tools for understanding the problems as they emerge and exist.

## Notes

1. Hereinafter “Wendt and Duvall (2008).”

2. The concept of the taboo is familiar to scholars of International Relations, at least in the form of the decades-long research interest into the “nuclear taboo” (e.g., Paul, 1995; Gizewski, 1996; Tannenwald, 1999; 2005; 2007; Fey et al, 2016; Dill et al, 2022).

3. This enforcement of total secrecy has shifted in recent years, although the substantial government resources devoted to managing official channels set up to disseminate information released around UFOs suggests that the issue management is still accorded a high priority from a good governance perspective. See, for example, *Preliminary Assessment: Unidentified Aerial Phenomena* https://www.dni.gov/files/ODNI/documents/assessments/Prelimary-Assessment-UAP-20210625.pdf

4. https://journals.sagepub.com/action/showMostReadArticles?journalCode=PTX, accessed March 6, 2023.

5. 50 U.S. Code § 3373 – Establishment of All-domain Anomaly Resolution Office; UAP Disclosure Act of 2023.

6. Tague-Sutcliffe also notes a potential impact on science policy.

7. Because the object of analysis is the corpus of citations, the methodological design does not permit the asking of questions about what should or could exist. Tables 1, 2, 3, and 4 suggest that there is a questionable foundation to claim that the article has been undercited in the context of peer items, placing the burden of proof for identifying the “dogs that didn’t bark” onto a future investigation with a different methodological design.

8. These included the eleven TRIPS 2007 journals—the *American Political Science Review,* the *American Journal of Political Science*, *World Politics*, *International Affairs, International Organization, International Studies Quarterly, Journal of Politics,* the *British Journal of Political Science,* the *Journal of Conflict Resolution*, *Security Studies,* and the *Journal of Peace Research*—as well as *International Security,* the *European Journal of International Relation,* the *Review of International Studies*, and *Millennium: Journal of International Studies*—selected for reputation.

9. www.scholar.google.com, collected 28 February 2023.

10. To facilitate the further exploration by more linguistically competent scholars, omitted articles were: Herborth (2011), Mo (2009, 2010, 2020); 劉正山 (2017).

11. https://www.scimagojr.com/, collected 1 March 2023.

12. See the discussion of journal selection in the methodology section above.

13. An adaptation of Latour’s suggestion to “follow the actors themselves” which he describes as the attempt “to catch up with their often wild innovations in order to learn from them what the collective existence has become in their hand, which methods they have elaborated to make it fit together, which accounts could best define the new associations that they have been forced to establish” (2005, 12).

14. While an interpretive scientometrics approach would be less directly relevant to the analysis of public discourse, we might consider how discussions around the UFO have been normalized through containment following the release of the Pentagon report. Certain authorities have the legitimacy to reveal new kinds of information, but speculation from amateurs remains stigmatized.
